# Microstructure Hierarchical Model of Competitive e^+^-Ps Trapping in Nanostructurized Substances: from Nanoparticle-Uniform to Nanoparticle-Biased Systems

**DOI:** 10.1186/s11671-017-1858-6

**Published:** 2017-01-25

**Authors:** Oleh Shpotyuk, Adam Ingram, Zdenka Bujňáková, Peter Baláž

**Affiliations:** 1 Jan Dlugosz University, al. Armii Krajowej, 13/15, 42201 Czestochowa, Poland; 2 Vlokh Institute of Physical Optics, 23, Dragomanov str., 79005 Lviv, Ukraine; 3 Opole University of Technology, 75, Ozimska str., 45370 Opole, Poland; 4 Institute of Geotechnics of Slovak Academy of Sciences, 45, Watsonova str., 04001 Košice, Slovakia

**Keywords:** Nanoparticles, Positron annihilation, Nanocomposite, Mechanochemical milling

## Abstract

Microstructure hierarchical model considering the free-volume elements at the level of interacting crystallites (non-spherical approximation) and the agglomerates of these crystallites (spherical approximation) was developed to describe free-volume evolution in mechanochemically milled As_4_S_4_/ZnS composites employing positron annihilation spectroscopy in a lifetime measuring mode. Positron lifetime spectra were reconstructed from unconstrained three-term decomposition procedure and further subjected to parameterization using x3-x2-coupling decomposition algorithm. Intrinsic inhomogeneities due to coarse-grained As_4_S_4_ and fine-grained ZnS nanoparticles were adequately described in terms of substitution trapping in positron and positronium (Ps) (bound positron-electron) states due to interfacial triple junctions between contacting particles and own free-volume defects in boundary compounds. Compositionally dependent nanostructurization in As_4_S_4_/ZnS nanocomposite system was imagined as conversion from o-Ps trapping sites to positron traps. The calculated trapping parameters that were shown could be useful to characterize adequately the nanospace filling in As_4_S_4_/ZnS composites.

## Background

Electron interaction with its antiparticle (positron) in lifetime measuring mode is known as an effective probing tool to characterize nanostructurization in solids possessing mixed trapping channels for annihilating positrons and their bound electron-positron states, i.e. positronium (Ps) atoms [1–5]. Intrinsic inhomogeneities due to *guest* nanoparticles (NP) of the same chemistry and size embedded in a structurally homogeneous *host* matrix (*NP-uniform composite* systems) can be adequately described in terms of substitution trapping in positron- and Ps-related sites, allowing estimation of interfacial voids or *triple junctions* (TJ) between contacting NP as free-volume elements (FVE) responsible for positron trapping and defect-free bulk lifetimes of nanostructurized matrix [6, 7]. Recently, this approach was proved for arsenic sulphide As_4_S_4_ NP capped with polyvinylpyrrolidone (PVP) as nonionic stabilizer to produce NP-uniform composite pharmaceuticals with pronounced anticancer activity [8]. Realistically, in many applications, the NP subsystem is modified with components of other nature to ensure additional functionality like fluorescent emission (ZnS, ZnSe, CdS, CdSe) and magnetically addressable drug delivery (Fe_3_O_4_). [9, 10]. In case of two different NP components forming *NP-biased composite* system, we apparently deal with high diversity of positron-Ps trapping paths resulting in complicated parameterization of responsible FVE.

In this work, we shall examine a hierarchical model of competitive positron-Ps trapping in composite system consisting of coarse-grained As_4_S_4_ and fine-grained ZnS NP.

## Methods

### Nanocomposite Preparation Procedure

The As_4_S_4_/ZnS nanocomposites were prepared by high-energy milling from commercial arsenic sulphide As_4_S_4_ (98%, Sigma-Aldrich, Germany) and some precursors taken for mechanochemical ZnS synthesis, the latter being zinc acetate, (CH_3_COO)_2_Zn · 2H_2_O (99%, ITES, Slovakia) and sodium sulphide, Na_2_S · 9H_2_O (98%, Acros Organics). The milling was performed in a 250-mL chamber with 50 balls (each 10 mm in diameter) made of tungsten carbide (WC) material using a planetary mill Pulverisette 6 (Fritsch, Germany). The whole treatment lasting 20 minutes was performed in protective Ar atmosphere under 500 rpm rotational speed of planet carrier. The sodium acetate obtained from the reaction was removed from products by washing with pure distilled water and, after drying, the solid As_4_S_4_/ZnS phase was obtained in different molar ratios (Table 1). Then, the powders were pelletized for further positron annihilation lifetime (PAL) measurements by compacting inside stainless steel die under ~0.7 GPa pressure, thus producing tablets having ~6 mm in diameter and ~1 mm in thickness.

**Table 1:** Estimated crystallite sizes in As_4_S_4_/ZnS nanocomposites

Molar ratio As_4_S_4_:ZnS	Crystallite size, nm	
	As_4_S_4_	ZnS
5:0	25	–
4:1	27	2.4
1:1	40	2.9
1:4	40	3.1
0:5	–	3.4

### Phase and Size Analysis

The crystallographical specificity of As_4_S_4_/ZnS nanocomposites was identified by X-ray diffraction method (Cu Kα_1_-radiation) employing D8 Advance diffractometer (Bruker, Germany). Mean sizes of arsenic sulphide As_4_S_4_ (JCPDS 01-072-0686) and sphalerite ZnS (JCPDS 01-0792) crystallites were estimated from the Rietveld refinement procedure as sizes of coherently diffracting domains in terms of isotropic line broadening [11]. As it follows from Table 1, these sizes are fitted to be in ~2.4–3.4 nm domain for ZnS crystallites and ~25–40 nm for As_4_S_4_ crystallites. Interestingly, the coarse-grained As_4_S_4_ crystallites apparently grow in size with the addition of fine-grained ZnS ones, this tendency being the most sharply revealed in As_4_S_4_/ZnS nanocomposites at high ZnS content.

### Free-Volume Structure Characterization

The PAL method was employed to study free-volume structure of As_4_S_4_/ZnS nanocomposites.

The raw PAL spectra of pelletized As_4_S_4_:ZnS nanocomposites were detected using fast-fast coincidence system ORTEC of 230 ps resolution (the full width at half maximum) based on two Photonis XP2020/Q photomultiplier tubes coupled to BaF_2_ scintillator 25.4A10/2M-Q-BaF-X-N detectors (Scionix, Bunnik, Holland) and ORTEC® electronics (ORTEC, Oak Ridge, TN, USA) [3]. The radioactive ^22^Na isotope of ~50-kBq activity wrapped by the Kapton® foil (DuPont^TM^, Circleville, OH, USA) and then sealed was used as a positron source sandwiched between two pellets. The normal-measurement statistics compressing 1 M annihilation events collected at stabilized measuring conditions was employed to ensure reliable PAL data. The 6.15-ps channel width allows a total number of channels to be 8000. Three separate measurements were performed for good reproducibility, the source contribution being evidenced at a level of 15% allowing full compensation of input from positrons annihilated in the Kapton® foil with a lifetime of 0.372 ns. The PAL spectra were fitted by three negative exponentials using LT 9.0 program [12], the errors in positron lifetimes *τ*_*i*_ and intensities *I*_*i*_ being ±0.005 ns and 0.5%, respectively. The annihilation channels were parameterized exploring formalism of unconstrained x3-term decomposition (under normalized component intensities *I*_*1*_ + *I*_*2*_ + *I*_*3*_ = 1.00), assuming separated contributions of positron trapping from one kind of defects (two-state trapping [1–3, 13, 14]) and Ps decaying through picking up an electron from the environment [1, 2, 13, 15]. Thus, the formalism of two-state positron trapping model [1–3, 13, 14] was utilized to parameterize mean *τ*_*av*_ and defect-free bulk *τ*_*b*_ lifetimes, as well as positron trapping rate in defects *κ*_*d*_, which was determined with ±0.01 ns^−1^ accuracy. In addition, the difference between defect-specific *τ*_*d*_ = *τ*_*2*_ and defect-free positron lifetimes (*τ*_*2*_–*τ*_*b*_) was taken as a signature of the size of positron traps in terms of equivalent number of vacancies, whereas the *τ*_*2*_/*τ*_***b***_ ratio was ascribed to the nature of these defects [1].

The Ps trapping formalism concerns positrons annihilating in porous substances as free particles or picking up an electron from the environment by forming a bound positron-electron state [1, 2]. In the ground state, the Ps atom exists as a singlet para-positronium (p-Ps) decaying intrinsically with two γ-quanta and a character lifetime in a vacuum of 0.125 ns, and triplet ortho-positronium (o-Ps) decaying with three γ-quanta and a lifetime of 142 ns. In matter, since the positron wave function is overlapping with the electrons outside, the annihilation with such electrons having an antiparallel spin decreases lifetime to 0.5–10 ns resulting in two γ-rays (“pick-off” annihilation) [2]. Two conditions should be satisfied to stabilize Ps, the first being sufficient size of free-volume void captured Ps and second being low electron density preventing direct positron-electron annihilation (that is why metals and semiconductors are excluded as potential Ps-forming media) [1, 2].

In respect to the known Tao-Eldrup formalism [1, 2], the localized Ps gives an indication on free-volume void radius *R* in terms of long-lived *τ*_*3*_ lifetime:

1 $$ {\tau}_{\mathbf{3}}=0.5\cdot {\left[1-\frac{R}{R+\varDelta R}+\frac{1}{2\pi}\cdot \sin \left(\frac{2\pi R}{R+\varDelta R}\right)\right]}^{-1}, $$

where Δ*R* = 0.166 nm is fitted empirical electron layer thickness [2].

The relative intensity of this component I_3_ correlates with density of Ps traps, giving fractional free volume *f*_*v*_ (in %) as

2 $$ {f}_v= C\cdot {V}_f\cdot {I}_3, $$

where *V*_*f*_ (in *Å* ^3^) is void volume in spherical approximation (4/3π*R* ^3^) and *C* = 0.0018 (as empirically determined constant for epoxy polymers [2]).

Doubtlessly, the above approach is meaningful under inessential input of the third component in the x3-decomposed PAL spectrum. However, this is not a case of NP-biased composites, where substitution trapping in positron and Ps sites is expected [4, 6, 7]. By ignoring nanostructurization without changing in trapping on a cost of full conversion from Ps sites to positron traps, we can describe the measured PAL data exploring *x3-x2-coupling decomposition algorithm* (x3-x2-CDA) [6–8]. Within this approach, we deal with x3-component PAL spectrum transformed to generalized x2-term form for *host* (initial) and nanostructurized *host-quest* (final) substances, where the second component involves contributions from all trapping channels (positron traps, o-Ps decaying and p-Ps self-annihilation). This allows resolving additional input with lifetime *τ*_*int*_ and intensity *I*_*int*_ in the second component of generalized x2-term PAL spectrum for *nanostructurized* solid, the compensating (*τ*_*n*_ *,I*_*n*_) input in the first channel being found from inter-channel equilibrium condition. Thereby, parameterization of substitution Ps-positron traps in nanostructurized solid can be performed by accepting *τ*_*n*_ *,I*_*n*_ and *τ*_*int*_ *,I*_*int*_ as respective components of x2-component PAL spectrum for hypothetical media strongly obeying the formalism of conventional two-state trapping model [1–3, 13, 14]. The defect lifetime *τ*_*int*_ in this model reflects the appeared/disappeared traps in respect to positive/negative sign of (*I*_*n*_ *, I*_*int*_) intensities.

## Results and Discussion

Expected positron-Ps trapping FVE in NP-biased composite systems are known to be defined by NP themselves (their chemical nature and geometrical specification), the interfacial free-volume defects or TJ with volume of a few missing atoms at the intersection of three or more grain boundaries forming main source for annihilating positrons [4, 6, 7, 13]. These TJ are highly diverse even for *NP-uniform composites*, being revealed within intra-, and inter-NP agglomerates [16]. When dealing with *NP-biased composites*, this diversity of positron-Ps trapping sites is expected to be substantially enhanced owing to different types of NP mixing and segregation [17].

Let’s examine expected positron-Ps traps in NP-biased coarse-fine-grained composite assuming a homogeneous physical mixture of two different solid NP, e.g. A (25–40 nm as for As_4_S_4_ crystallites produced by milling from bulk precursors, see Table 1), and B (2.4–3.4 nm as for ZnS crystallites produced by milling from chemically synthesized precursors, see Table 1).

Noteworthy, *the bottommost hierarchical level* of VFE in NP-biased composites comprising inter-crystalline interactions is composed of vacancy-type defects (multi-vacancies) in “pure” A and B components and inter-crystalline TJ of irregular shape in view of rather non-spherical approximation validated for such crystallites. The agglomerated homogeneous (A and/or B) or inhomogeneous A-B close-packed crystallites serve as *precursors* for composite-forming NP.

At the next level, *the uppermost hierarchical level* of FVE, we adopt interactions between agglomerated loosely packed crystallites to form distinct NP in a composite system. Spherical approximation to NP themselves is accepted. At the same time, for A-B mixture, we assume the constituent segregation under the competitive content of A and B components (close to 1:1composites) or the preferentially ordered unit segregation approaching boundary A and B compositions (5:0 or 0:5) [17, 18]. These prerequisites can be reasonably justified for As_4_S_4_/ZnS nanocomposite affected by high-energy milling provided in a dry mode [8, 9].

**Fig. 1:**
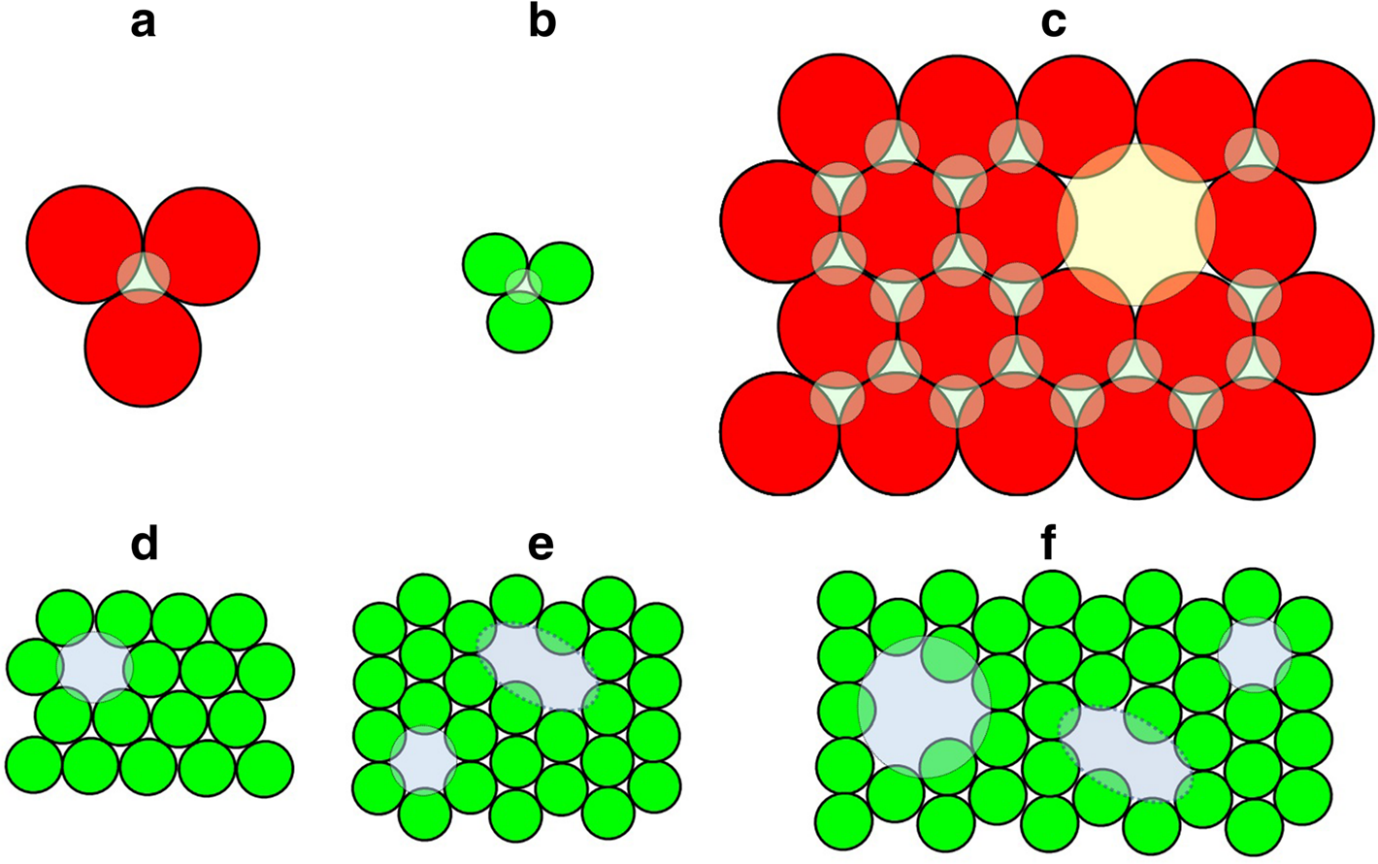
FVE in mixed coarse-fine-grained A-B composite system showing interfacial TJ in purely homochemical A+A+A (**a**) and B+B+B (**b**) environment, as well as vacancy-type voids in A- (**c**) and B-subsystems (**d–f**)

**Fig. 2:**
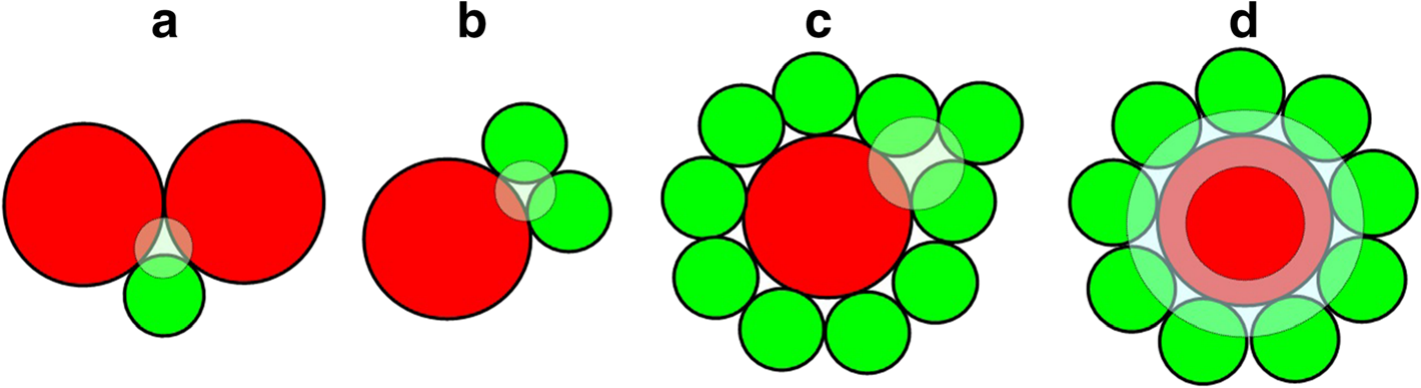
FVE in mixed coarse-fine-grained A-B composite system showing interfacial TJ in heterochemical A-preferential A+A+B (**a**), B-preferential A+B+B (**b**), A+B+B+B (**c**) and grain-boundary A+nB (**d**) environment

Both boundary 5:0 and 0:5 composites form interfacial TJ in purely homochemical A- and B-environment, respectively, depicted on Fig. 1a, b. Within this approach, of three hard-contacting spheres of *R* radius, such interfacial TJ can be roughly imagined as equilateral triangles with close to *R* side. For mixed A-B composites, these TJ attain A-, or B-preferential heterochemical environment as shown on Fig. 2. With going from coarse-grained A (5:0) to fine-grained B (0:5) composites, the homochemical A-type TJ (Fig. 1a) are gradually replaced by heterochemical A- and B-preferential TJ, as shown respectively in Fig. 2a–d, so B-rich nanocomposites demonstrate higher diversity of expected TJ.

As to own vacancy-type defects, these voids are not important for positron-Ps trapping in A-subsystem (Fig. 1c) in view of overestimated open volumes (a few thousands of nm^3^), which are far beyond the measuring limits of PAL spectroscopy [1–3]. In contrast, the FVE in a form of vacancies in B-subsystem (Fig. 1d–f) are more PAL-sensitive, enhancing trapping rate in B-rich composites. With accepting irregularity and consequently more closer packing in space arrangement of these B NP, the volumes of corresponding trapping sites are expected to be essentially less than those geometrically regular shown in Fig. 1.

**Fig. 3:**
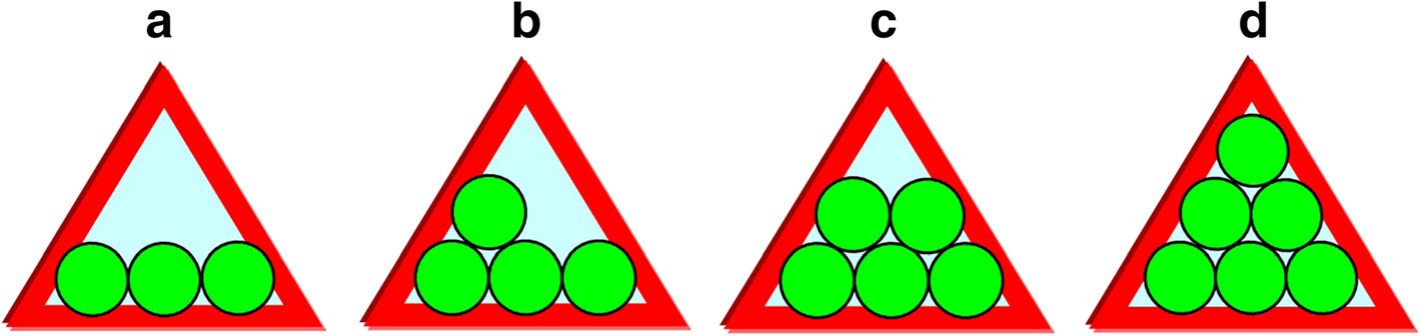
An amplified cartoon view showing FVE in interfacial TJ of coarse-grained A-subsystem (*red-distinguished triangles*) due to occupancy with fine B NP (*green-colored*).

Finally, the third kind of FVE that is meaningful for mixed positron-Ps trapping within this *hierarchical model* is realized in A-B composites due to TJ in coarse-grained A-subsystem filled with fine B NP (Fig. 3). This channel can be validated only under essential difference in NP sizes, especially when Ps trapping TJ in A-subsystem (red-distinguished by large triangles in Fig. 3) are reduced in volume due to embedded B NP, thus producing effective positron trapping sites. Spherical-like approach to these FVE allows their simple separation on distinct components contributing to different positron-Ps trapping channels, while expected volumes themselves can be essentially disturbed in realistic composites owing to more irregular shape of NP. As a rule, shape irregularity causes denser packing of contacting NP resulting in underestimated void volumes. In the first hand, this concerns interfacial TJ between coarse-grained crystallites in A-subsystem, which possess gradually less free volumes than those assuming hard-contacting spheres. In reality, the expected volumes of these TJ will be somewhat depressed due to amorphous phase present after high-energy milling. Thus, it means that all estimated free volumes should be accepted as the upper limits in a mixture of hard A-B spheres forming a realistic nanoparticle-biased composite system.

FVE evolution in As_4_S_4_/ZnS nanocomposites tested with PAL spectroscopy confirms this model proposed for NP-biased coarse-fine-grained A-B composite system.

**Fig. 4:**
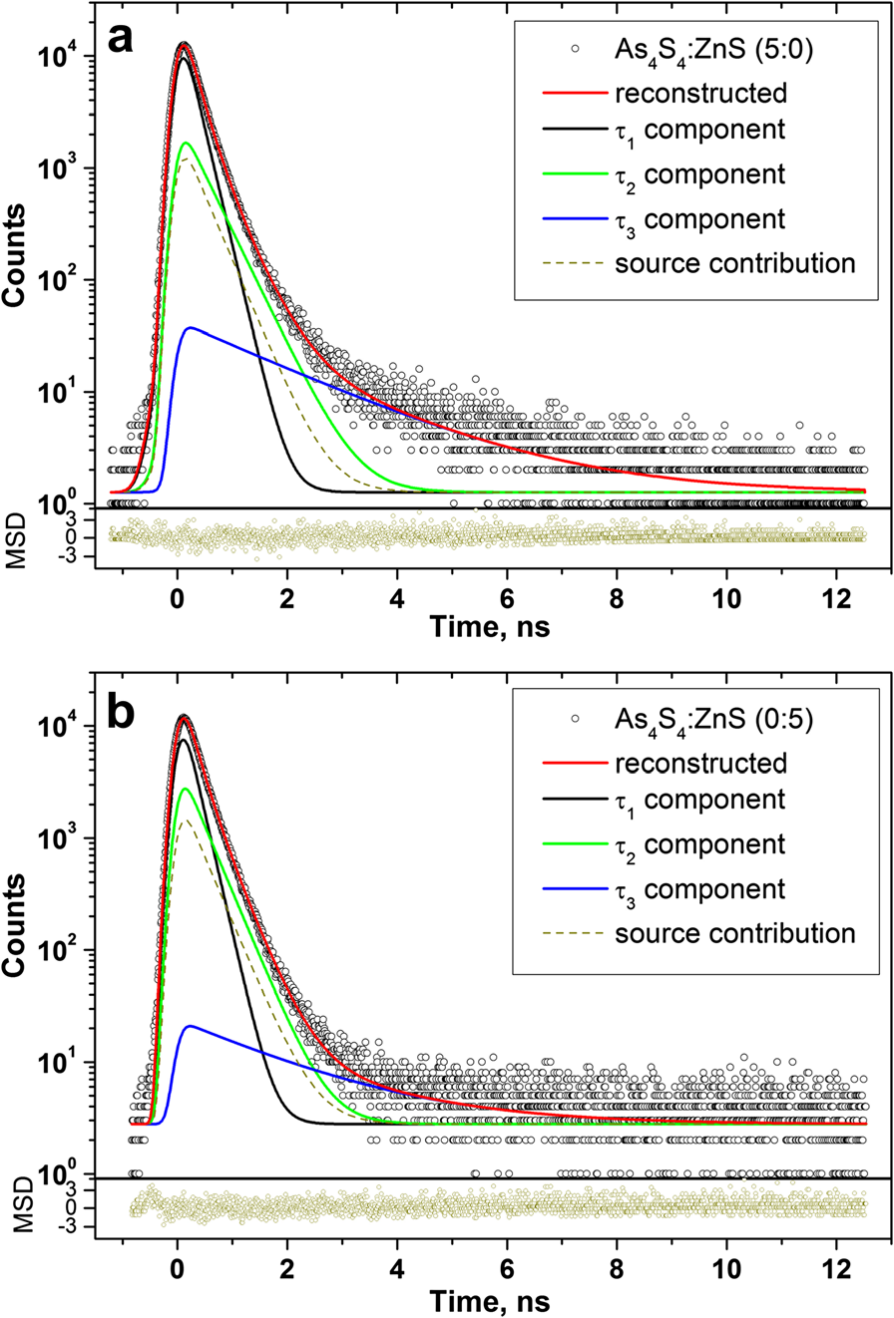
PAL spectra of boundary 5:0 (**a**) and 0:5 (**b**) representatives of pelletized As_4_S_4_/ZnS nanocomposites reconstructed from x3-fitting procedure at the background of source contribution (*bottom inset* shows statistical scatter of variance; channel width of PAL measurements is 50 ns)

**Fig. 5:**
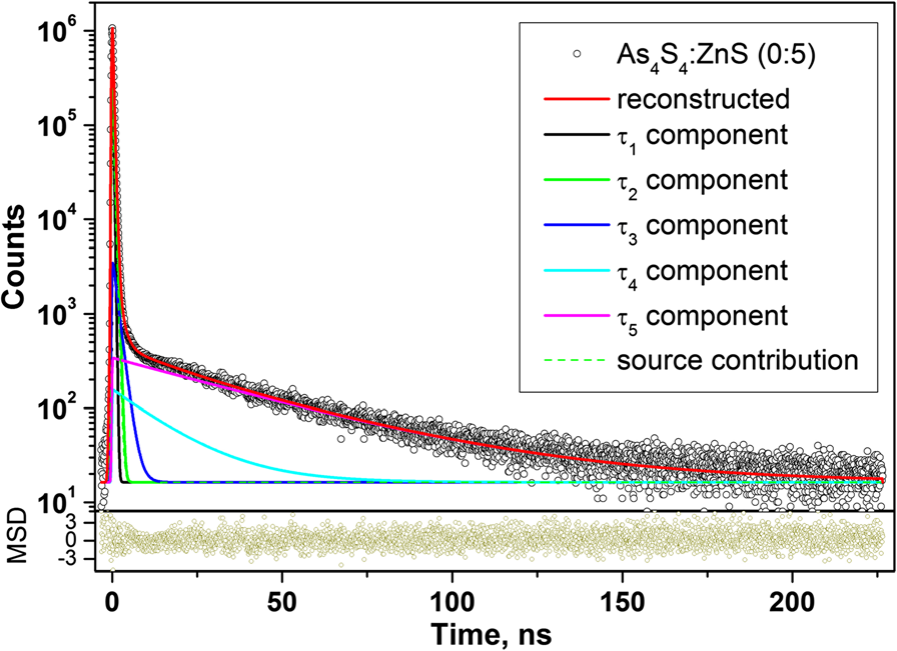
PAL spectra of 0:5 (ZnS) nanocomposites reconstructed from unconstrained x5-fitting procedure at the general background of source contribution (*bottom inset* shows statistical scatter of variance, channel width of PAL measurements is 500 ns)

The raw PAL spectra registered under channel width of 50 ns for boundary 5:0 and 0:5 specimens of pelletized As_4_S_4_/ZnS nanocomposites reconstructed from x3-term fitting procedure are shown in Fig. 4, corresponding the best fit positron and Ps trapping modes being given in Table 2. The similar spectra were detected for all intermediate As_4_S_4_/ZnS nanocomposites (4:1, 1:1, 1:4). The narrow values of statistical scatter of variance tightly grouped around 0-axis testify that PAL measurements are adequately described by this fitting procedure, except a 0:5 sample composed entirely of ZnS NP. In this latter case, it was possible to decompose the PAL spectrum (see Fig. 4b) on four or five unconstrained components under channel width of 500 ns (see Fig. 5) without essential decrease in goodness of fitting procedure, the results of such decomposition being presented in Table 3. This testifies in favour of many Ps trapping channels in low-sized ZnS nanocomposites due to possible input from FVE of the bottommost hierarchical level (vacancy-type defects and inter-crystalline TJ) and multivacancy voids in ZnS crystallite packing (as those shown in Figs. 1f , 2c, d). It should be noted that character sizes of o-Ps trapping voids estimated in a spherical approximation using Eq. 1) are well fitted to *R* ≅ 0.27–0.30 nm with free-volume fraction *f*_*v*_ ≅ 0.14–0.20% (Table 2). In purely monoparticle ZnS-based composite (0:5 composite), the contribution of larger o-Ps trapping sites with radii *R* ≅ 12–14 nm and *f*_*v*_ ≅ 43–44% (Table 3) is more essential.

**Table 2:** Fitting parameters and PAL trapping modes describing positron annihilation in pelletized As_4_S_4_/ZnS nanocomposites (the channel width of PAL measurements is 50 ns)

Composite As_4_S_4_:ZnS	PAL spectra fitting parameters					Positron trapping modes				Ps trapping modes	
	*τ*_*1*_	*τ*_*2*_	*τ*_*3*_	*I*_*2*_	*I*_*3*_	*τ*_*b*_	*κ*_*d*_	*τ*_*2*_ *-τ*_*b*_	*τ*_*2*_ */τ*_*b*_	*R*	*f*_*v*_
	ns	ns	ns	a.u.	a.u.	ns	ns^−1^	ns	a.u.	nm	%
5:0	0.209	0.433	2.089	0.212	0.010	0.235	0.53	0.20	1.84	0.296	0.19
4:1	0.202	0.399	1.856	0.250	0.011	0.231	0.61	0.17	1.73	0.275	0.17
1:1	0.202	0.387	1.705	0.288	0.015	0.235	0.69	0.15	1.65	0.259	0.20
1:4	0.194	0.378	1.804	0.286	0.013	0.226	0.73	0.15	1.68	0.269	0.19
0:5	0.185	0.375	1.955	0.341	0.008	0.224	0.94	0.15	1.67	0.284	0.14

**Table 3:** PAL spectra parameterization of pelletized 0:5 nanocomposites (formed of pure ZnS) under channel width of 500 ns employing unconstrained x4- and x5-fitting procedures

PAL spectra fitting parameters									Ps trapping modes					
Lifetimes, ns					Intensities, %				*R*_*3*_	*f*_*3*_	*R*_*4*_	*f*_*4*_	*R*_*5*_	*f*_*5*_
*τ*_*1*_	*τ*_*2*_	*τ*_*3*_	*τ*_*4*_	*τ*_*5*_	*I*_*2*_	*I*_*3*_	*I*_*4*_	*I*_*5*_	nm	%	nm	%	nm	%
0.192	0.400	2.092	37.06	–	0.41	0.008	0.029	–	0.296	0.16	1.262	44.2	–	–
0.183	0.360	1.473	15.41	42.34	0.51	0.010	0.004	0.023	0.233	0.10	0.827	1.86	1.354	43.0

Before detailed analysis of the PAL data measured, it is important to recognize existing positron-electron annihilation paths in pure counterparts of the studied As_4_S_4_/ZnS nanocomposites.

The coarse-grained component, the arsenic sulphide As_4_S_4_, exists at least in three crystalline polymorphs, these being low-temperature α-As_4_S_4_ structurally identical to mineral realgar, high-temperature β-As_4_S_4_ and pararealgar as an alteration product from both α- and β-phases [19, 20]. All polymorphs are built of cage-like As_4_S_4_ molecules filling a space to form denser (~14.8 Å^3^ per molecule in realgar) or looser structural arrangement (~15.7 Å^3^ per molecule in pararealgar) [20]. Reliable PAL measurements for realgar testify on defect-free bulk lifetime *τ*_*b*_ ≈ 0.223 ns and defect lifetime*τ*_*d*_ = *τ*_*2*_ ≅ 0.346 ns due to positron traps with ~80 Å^3^ volumes character for tri- and tetra-atomic vacancies (such traps are overlapped low electron-density spaces around S atoms forming As_4_S_4_ cage molecules) [19]. Because of similar covalent bonding and space filling efficiency in all As_4_S_4_ polymorphs, it seems reasonable a close proximity between corresponding positron traps.

The fine-grained component, the zink sulphide ZnS belonging to II–VI group compound wide band-gap semiconductors, exists in the form of hexagonal *wurtzite* and cubic *zink blende* [21]. Whichever crystal preparation technology, this material demonstrates bulk positron lifetimes *τ*_*b*_ ranging within 0.215–0.230 ns domain [22–24] (in good accordance with theoretical calculations [25]), vacancy-related components (0.266 ns for monovacancy and 0.286 ns for divacancy [22]), and longer lifetime of 0.430 ns attributed to voids or grain boundaries [23].

As seen from Table 2, the bulk positron lifetimes *τ*_*b*_ for boundary 5:0 and 0:5, composites are remarkably close to those characters for realgar, α-As_4_S_4_ (0.223 ns) and ZnS polycrystals (0.230 ns), testifying that evolution of FVE occurs in direct chemical environment of these crystalline species. At the same time, the defect-related lifetimes *τ*_*d*_ = *τ*_*2*_ are essentially higher than those character for vacancy-type defects in these crystals (0.342 ns for α-As_4_S_4_ [19] and 0.266–0.286 ns for ZnS [22]), thus meaning that other types of free-volume defects are essential in both subsystems. These defects are apparently interfacial TJ between agglomerated nanocrystallites as it characterize for other similar nanostructurized substances [4, 6–8]. With going from 5:0 (As_4_S_4_) to 0:5 (ZnS) composites, a growing tendency is observed in positron trapping rate *κ*_*d*_ due to increase in the content of these defects (due to accompanying growing trend observed in *I*_*2*_ intensity, see Table 2). In contrast, the o-Ps trapping modes are rather in an opposite compositional dependence, showing decrease in *I*_*3*_ intensity accompanied by increase in *τ*_*3*_ lifetime towards both boundary compositions (5:0 and 0:5) in respect to central 1As_4_S_4_:1ZnS composite. The main void-evolution process governing the behaviour of the third component in x3-term decomposed PAL spectra (Table 2) can be imagined as a contribution from interfacial TJ caused in coarse-grained As_4_S_4_-subsystem due to occupancy with fine-grained ZnS NP (Fig. 3). Additional input to Ps trapping channel in As_4_S_4_/ZnS nanocomposites is expected for high content of agglomerated ZnS NP due to multivacancy voids (see Fig. 1f).

Hence, the nanostructurization in As_4_S_4_/ZnS composite system can be indeed compositionally imagined as conversion from o-Ps trapping sites to positron traps, thus allowing x3-x2-CDA formalism [6–8] to analyze the measured PAL spectra. The corresponding NP-related PAL trapping modes resulting from such treatment are given in Table 4.

**Table 4:** NP-related PAL trapping modes in pelletized As_4_S_4_/ZnS nanocomposites treated within x3-x2-CDA in respect to 1As_4_S_4_:1ZnS composite

Composite samples As_4_S_4_:ZnO	I component		II component		PAL trapping modes				
	*τ*_*n*_	*I*_*n*_	*τ*_*int*_	*I*_*int*_	*τ*_*av*_	*τ*_*b*_	*κ*_*d*_	*τ*_*2*_ *-τ*_*b*_	*τ*_*2*_ */τ*_*b*_
	ns	a.u.	ns	a.u.	ns	ns	ns^-1^	ns	a.u.
5:0	0.244	0.127	0.875	0.020	0.330	0.271	0.40	0.604	3.23
4:1	0.202	0.133	0.464	0.039	0.261	0.231	0.63	0.233	2.01
1:4	0.145	0.099	0.316	0.036	0.191	0.170	1.00	0.146	1.86
0:5	0.172	0.373	0.365	0.187	0.237	0.209	1.02	0.156	1.74

As it follows from analysis of x3-x2-CDA modes for boundary 5:0 and 0:5 nanocomposites, unique As_4_S_4_-related trapping sites are rather o-Ps traps with *τ*_*int*_ = 0.875 ns, which can be ascribed (due to bulk positron lifetime *τ*_*b*_ ≈ 0.271 ns essentially increased in respect to *τ*_*b*_ ≈ 0.223 ns proper for realgar α-As_4_S_4_ [19]) to interfacial TJ in a random network of loose-packed As_4_S_4_ NP (see Fig. 1a). In contrast, the ZnS-related trapping sites are typical positron traps with a lifetime of *τ*_*int*_ = 0.365 ns, which can be attributed (due to proximity in *τ*_*b*_ ≈ 0.209 ns to bulk positron lifetimes of crystalline ZnS [22–24]) to multivacancy clusters in a network of more close-packed ZnS NP (Fig. 1d–f) and free-volume voids in preferential ZnS environment (Fig. 2c, d). The latter free-volume defects are dominant in ZnS-rich nanocomposites along with defect-related monovacancy (*τ*_*d*_ = 0.266 ns) and divacancy (*τ*_*d*_ = 0.286 ns) trapping sites. In As_4_S_4_-rich 4:1 nanocomposites, the preferential traps are interfacial Ps trapping TJ filled with fine-grained ZnS NP (Fig. 3).

The whole microstructure-hierarchical model illustrating compositional diversity of interchangeable positron-Ps trapping sites in coarse-fine-grained As_4_S_4_/ZnS NP-biased composites is shown in Table 5. The interfacial TJ in homochemical As_4_S_4_ and ZnS environment along with multivacancy defects in fine-grained ZnS subsystem are shown to be the governing FVE in “pure” boundary composites. The greatest variety of positron-Ps trapping paths owing to interfacial TJ in mixed heterochemical As_4_S_4_-ZnS environment is expected for 1As_4_S_4_:1ZnS nanocomposite.

**Table 5:**
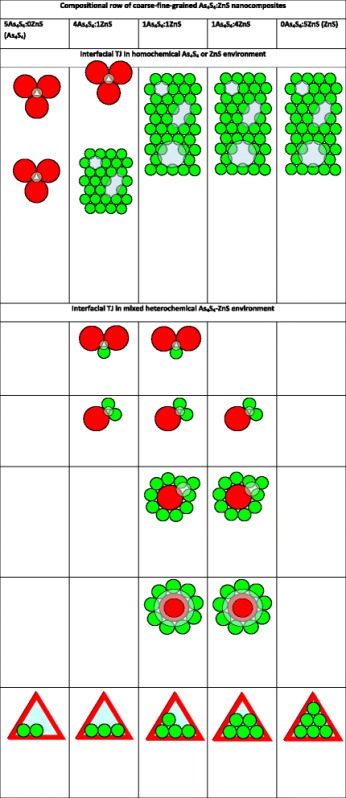
Microstructure-hierarchical model showing compositional diversity of interchangeable positron-Ps trapping sites in coarse-fine-grained As_4_S_4_/ZnS nanocomposites (*bottom row* represents FVE in interfacial TJ of coarse-grained As_4_S_4_-system due to occupancy with fine-grained ZnS NP)

## Conclusions

The method of annihilating positrons in the lifetime measuring mode is employed to study competitive positron-Ps trapping channels in nanoparticle-biased physical mixtures exemplified by coarse-fine-grained As_4_S_4_/ZnS nanocomposites prepared by high-energy milling.

Positron lifetime spectra are reconstructed from unconstrained three-term decomposition and then subjected to parameterization using x3-x2-coupling decomposition algorithm. To separate eventual contributions in mixed positron-Ps trapping channels, the microstructure-hierarchical model considering free-volume elements in nanocomposites at the level of interacting crystallites (non-spherical approximation) and agglomerates of crystallites (spherical approximation) is developed. Assuming a model of hard-contacting spheres for both coarse-grained As_4_S_4_ and fine-grained ZnS nanoparticles in different preferential chemical environments, the main void-evolution process governing the behaviour of the third component in three-term decomposed positron lifetime spectra is identified as a contribution from interfacial triple junctions in coarse-grained As_4_S_4_-subsystem due to occupancy by fine-grained ZnS nanoparticles. The defect-formation processes in coarse-fine-grained As_4_S_4_/ZnS nanocomposites are shown to occur in homochemical environment of more compact fine-grained ZnS nanoparticles inserted in looser coarse-grained As_4_S_4_ environment. Trapping parameters calculated within x3-x2-coupling decomposition procedure are shown to characterize adequately nanospace filling in As_4_S_4_/ZnS nanocomposites.

## Abbreviations

CDA: Coupling decomposition algorithm
NP: Nanoparticles
PAL: Positron annihilation lifetime
TJ: Triple junction
